# Encyclopædia Medica

**Published:** 1902-12

**Authors:** 


**Encyclopædia Medica.** Under the General Editorship of CHALMERS WATSON, M.B. Vol. X. Pregnancy to Scarlet Fever, Pp. vi., 516. Vol. XI. Sciatica to Syncope, Pp. vi., 530. Edinburgh: William Green & Sons. 1902.

Two further volumes of this great work, previously noticed by us (*vide* Vols. xvii., xviii., and xx.), are bringing it near to a completion. Two articles, Pregnancy and Puerperium, form a large part of the tenth volume, and it is in accordance with the fitness of things that much space should be devoted to the consideration of these processes in which we have all played an important part.

Complications of pregnancy are apt to be overlooked by the indifference with which most of us regard that which is too often considered to be a mere physiological incident, and it is well that doctors should be reminded of the desirability of a much more systematic enquiry into the pregnant woman's condition than is usually practised. The array of possibilities here recorded is truly appalling, and might easily and unnecessarily alarm the tyro in the art if he is not aware that many of the conditions described have little interest beyond that which is academic and curious. In the same paragraph in which Dr. John Phillips states that "the presence of albumin to a small extent is found in a considerable proportion of pregnant women" it is a relief to find that the "considerable" proportion is 2 to 4 per cent. In the description of the oncoming of symptoms of albuminuria there is, associated with a scientific distinction of para-globulin and serum-albumin, a touch of pathos—"a working-woman will often complain of inability to thread her needle." Is this symptom limited to persons in the class of life mentioned? If so, it seems to be a specially hard dispensation of Nature, as the working-woman will probably have more difficulty in preparing for the coming event than one who is more fortunately placed in life so far as this world's goods are concerned. The treatment of the commoner forms of difficulty is well described. The writer, whilst dogmatic as to the line he himself follows, states very fairly the reasons which lead others to adopt a different course.

It is difficult to see on what grounds Dr. A. E. Giles, in his article on the Physiology of the Puerperium, recommends a vaginal douche to correct the offensiveness of the lochia. If any irrigation is necessary it should be applied to the source of the trouble, and not merely to the channel through which the discharge passes. Dr. Giles rightly considers that too much importance is often attached to immediate intra-uterine douching. Only in very exceptional cases is it necessary, and the uterus and its owner are all the better for rest after labour is over. To correct any trouble which may arise from absorption from laceration of the vagina or its outlet we find that nothing is better than the introduction of a pessary of twenty grains of iodoform night and morning for the first three days and once a day afterwards for a week. The fear of opium for after-pains Dr. Giles unduly magnifies. His observations about diet are particularly sane; but one wonders why "the patient may be transferred to a couch as early as the tenth day," when he says that "it is an advantage for her to remain in bed for a full fortnight." Thirty-two pages are devoted by Drs. W. J. Smyly, J. Haig Ferguson, W. Fordyce, and G. R. Wilson, to the consideration of the Pathology of the Puerperium, and all should be studied with care. Much was expected from the use of anti-streptococcic serum, but Dr. Smyly states that "the majority of authorities have reported unfavourably upon it."

In the bibliography it would have been more satisfactory if the names of the writers had always been given. For instance, in that of Diseases of the Fœtus, the name of Dr. Ballantyne is not mentioned, although reference is made to many of his valuable communications.

Various articles on skin diseases form a large part of the eleventh volume.

The article on Sclerema Neonatorum attempts to clear up the confusion which exists between this affection and Œdema Neonatorum—both extremely rare in this country. They both occur at or soon after birth; but in the former there seems to be no pitting on pressure, in the latter there is. They both consist of induration of the connective tissues: the first is invariably fatal, the second not quite so hopeless. They both point to great depression of the vital organs.

Sclerodermia, again, of which the keloid of Addison is one variety, presents many symptoms resembling the two above affections; but these cases, although they may occur in early life, do not affect infants under 13 months.

The earliest case of Scleroma occurred at the Stockholm Hospital, and is related by Usenbenzius (A.D. 1718). The child was born at eight months—the midwife said—alive, but died soon afterwards. The description of the case had the graphic title—"Fœtus vivus, frigidus et rigidus."

The "Anatomy and Physiology" of the Skin are comprised in twelve concise and well-arranged pages. The six following pages, on "Bacteriology of the Skin," contain about everything that is known on this subject; but a great deal of this can only be conjectural. No doubt a great many germs are specific, but a great many are only the residents on eligible breeding-grounds. Yaws affords a good example (p. 142): "Several micro-organisms have been isolated in association with the lesions of Yaws, and have been regarded as specific by those who have described them; but specific characters have not been definitely established. Among these are a *yeast* (Powell), a *bacillus* (Breda), and a micro-coccus (Nicholls and Watts).

Regarding the diagnosis of Lupus, we can speak with great confidence of the value of the diascopic method of Unna. This glass-pressure of the skin—with varying appliances—is unquestionably of great service. "Kerion" of the upper limbs in adults—rare affection, but to be noted—seems to be produced by the tricophyton megalosporon ectothrix, which is a pus producer on its own account, and therefore needs no pus cocci. Tinea nodosa is well described—a rare affection of the hairs of the moustache and beard—the spores are arranged at right angles to the hair shaft. Melanin, according to Leslie Roberts, on Pigmentary Diseases of the Skin, plays a very important part. It consists largely of carbon—and is free from iron—and herein differs from hæmo-globin. Facts favour the idea that the epithelium is a complementary organ to the pigment secreting supra-renal organs. The remedy for Leucoderma, if there is one, has still to be found. Skin-grafting and allied proceedings are well and concisely dealt with.

Smallpox (pp. 212—238), by Dr. Hope, Medical Officer of Health for Liverpool, is well described, without being too verbose, and the photos, temperature charts and tables are useful; but there is little said about the hemorrhagic form. It certainly does seem that the poison of this disease is perhaps more contagious than any other infectious disease, and it persists after life has ceased — the cadaver unquestionably contains or exhales the virus.

In the article on Snake-Bites, the venom of the cobra is described, and the sp. gravity of it stated to be 10.50; surely this must be an error in printing, 1050 being meant.

An interesting article by Dr. Hyslop, on Sleep and Somnambulism, is followed by one on Sleeping Sickness or Negro Lethargy. This curious malady is described by Dr. P. Manson, C.M.G.

